# Postnatal Depression and Post-Traumatic Stress Risk Following Miscarriage

**DOI:** 10.3390/ijerph19116515

**Published:** 2022-05-27

**Authors:** Milda Kukulskienė, Nida Žemaitienė

**Affiliations:** Department of Health Psychology, Faculty of Public Health, Lithuanian University of Health Sciences, 44307 Kaunas, Lithuania; nida.zemaitiene@lsmuni.lt

**Keywords:** miscarriage, women’s wellbeing, postpartum depression, post-traumatic stress

## Abstract

The experience of miscarriage is an important population-level problem that affects approximately 10–25% of pregnancies. The physical consequences of miscarriage have been researched extensively, but psychological sequelae less so. First-person accounts show that women who have experienced miscarriage feel pressured to stay silent, to grieve, and to fight intense physical and psychological challenges alone. There is ample scientific evidence on the links between miscarriage and physical and mental health disorders, such as complicated grief, anxiety, depression, post-traumatic stress, suicidal risk, psychosomatic disorders, sexual health disorders, etc. However, there is a lack of deeper understanding of the specifics of psychological morbidity after miscarriage, as well as of the information on vulnerability and resilience factors. This study aims to assess the risk of postnatal depression and post-traumatic stress following miscarriage. A total of 839 Lithuanian women who had one or more miscarriages were asked to complete an online questionnaire, including the Edinburgh Postnatal Depression Scale (EPDS) and the Impact of Events Scale-Revised (IES-R). Of the women, 59.1% were found to be at increased risk of postnatal depression and 48.9% at high risk of postnatal depression; 44.7% of the women were considered to be at increased risk of post-traumatic stress. An impaired relationship with one’s body and childlessness has been the strongest predictors of psychological morbidity risk.

## 1. Introduction

The definitions of pregnancy loss are inconsistent around the world, whereas the terms miscarriage and spontaneous abortion are used interchangeably [1,2]. According to the World Health Organization (WHO), pregnancy loss before 28 weeks of pregnancy should be referred to as miscarriage and pregnancy loss at or after 28 weeks as a stillbirth [3]. However, such a distinction is contradictory. For example, in another source, the WHO defines spontaneous abortion as loss of pregnancy before fetal viability up to 22 weeks of gestation [4], and, according to the guidelines of different organizations in Europe, the UK, and the US, miscarriages are classified as pregnancies that ended before 20–24 completed gestational weeks [5,6,7,8,9].

The experience of miscarriage is an important population-level problem. It affects approximately 10–25% of pregnancies [1,3,10]. However, it is difficult to systematically record miscarriage statistics, especially in cases of early pregnancy loss. Members of society who have no personal history of pregnancy loss lack knowledge and often misunderstand basic reproductive health information related to miscarriages [11]. It should be emphasized that the physical consequences of miscarriage have been researched extensively, but psychological sequelae less so [1]. Loss of pregnancy still remains a heavily stigmatized taboo subject, and its impact on a woman’s physical and mental health has been greatly underestimated. First-person accounts around the world show that women who have experienced miscarriage feel pressured to stay silent, to grieve, and to fight intense physical and psychological challenges alone [3].

This reproductive health question is highly complex, as it affects not only women’s health but also their partners, children, and the whole family [12]. There is ample scientific evidence on the links between miscarriage and physical and mental health disorders, such as complicated grief, anxiety, depression, post-traumatic stress, suicidal risk, psychosomatic disorders, sexual health disorders, etc. [12,13,14,15,16,17,18,19,20,21,22,23,24]. One of the largest studies on longitudinal morbidity after pregnancy loss has shown that one month after loss of pregnancy, 29% of women revealed symptoms suggestive of post-traumatic stress, 24% of moderate–severe anxiety, and 11% of moderate–severe depression. Despite the fact that these symptoms declined over time, they remained at clinically important levels even after nine months [18]. Other researchers have found that moderate to high depression risk was prevalent in more than a third of women one month after miscarriage (34.1%); thoughts of self-harm were observed in 33.1% of women [23]. In several studies, the prevalence of PTSD has been estimated to be 25–29%, with symptom severity similar to other traumatized populations [15,16]. Another study found that 43.9% of women after miscarriage reported clinical levels of post-traumatic stress symptoms [20]. Additionally, women’s partners are also known to be at significant risk of anxiety, depression, and PTSD symptoms, but to a lesser extent [17]. Recent studies highlight the importance of noticing early symptoms of postnatal depression, as it can last for years if left untreated [25]. It is emphasized that emotion-aware smart systems could be applied for more effective psychological morbidity prediction and prevention, as well as sentiment analysis, which could be useful for monitoring high-risk pregnancies [25,26].

However, there is a lack of deeper understanding of the specifics of psychological morbidity after miscarriage, as well as of the information on vulnerability and resilience factors. Opinions as to which demographic and miscarriage-related aspects are the most important underlying factors for postnatal depression and post-traumatic differ. Based on just a few literature sources, the key drivers to psychological morbidity after miscarriage include demographic factors such as younger age, lower education level, history of psychiatric illness, lack of social support, being single or poor marital adjustment, and pregnancy- or miscarriage-related characteristics, i.e., previous pregnancy loss(es), mode of conception, ambivalence towards a fetus, later gestational week, induced miscarriages, surgical interventions, etc. [12,13,22,23,24]. It has been determined that symptoms of depression and perinatal grief tend to decrease in 9 months but only for women with children and for those who were satisfied with healthcare services [14]. Childlessness and insufficient postnatal healthcare might constitute those risk factors, which are typically associated with poor adjustment.

Most of the studies that analyzed women’s well-being and psychological morbidity after miscarriage were conducted in the United States, Western Europe, and Scandinavian countries. However, it is difficult to predict what results should be expected in the Lithuanian sample, as the research data vary a lot. There is not enough data on the actual prevalence of pregnancy losses in Lithuania, as well as on physical and psychological reactions of women and their partners, on postnatal healthcare, and the need for social support. It is yet unknown which factors postnatal and mental healthcare specialists should focus on in order to screen and prevent psychological morbidity after prenatal losses more effectively. Therefore, this study aims to assess the risk of postnatal depression and post-traumatic stress following miscarriage.

## 2. Materials and Methods

This publication presents a cross-sectional study on women’s well-being in relation to previous miscarriage(s). The procedures for data collection, analysis, and storage were approved by the Kaunas Regional Biomedical Research Ethics Committee (No. BE2-99, 23 October 2019; No. P1-BE-2-99/2019, 15 December 2021). The scientists observed all the ethics guidelines for data collection and information processing.

This study is a part of an ongoing Ph.D. thesis project “Miscarriage Experience in Women: The Process of Surviving Foetal Loss, Coping, and Needs of Assistance”. A quantitative study was implemented in order to validate the qualitative findings of the first project’s stage. A part of the results of the qualitative interview analysis was presented in the previous publication [27]. 

The data collection was initiated in December 2021 and was completed in February 2022. The electronic survey was uploaded online and was available for about three months. The invitation to participate in the study was publicized in the target social networking groups, pages, and blogs, encompassing the topics of pregnancy, motherhood, and reproductive challenges, such as miscarriages, infertility, loss of a baby, etc. The invitation was shared by more than 100 social media users and pages.

A total of 839 Lithuanian women who had experienced a single (n = 559) or recurrent miscarriage(s) (n = 280) in their medical history participated in the study. The age of the participants ranged from 19 to 55 years (a mean of 33.34 years, SD 5.46). The demographic and miscarriage-related characteristics are provided in Table 1. The participants were also asked to indicate the year when their last miscarriage occurred. The indicated dates ranged from 1993 to 2022. A total of 204 participants had experienced their last miscarriage in 2021–2022. In calculations participants who had experienced miscarriage in 2021–2022 years were excluded to check whether the past period is relevant to the manifestation of the EPDS and PTSD symptoms. As the results did not differ significantly, it was decided to include recent miscarriage experiences in further calculations as well.

The questionnaire consisted of the following six thematic blocks (95 statements in general): A. Demographic and miscarriage-related characteristics; B. Emotional and physical well-being after miscarriage (well-being before pregnancy, immediately after miscarriage, and one month after miscarriage, and changes in the relationship with one’s body); C. Edinburgh Postnatal Depression Scale (EPDS) (Cronbach’s alpha—0.89); D. Impact of Events Scale-Revised (IES-R) (Cronbach’s alpha—0.95); E. Questions about coping after miscarriage; and F. Questions about the needs for assistance and support. Validated and adapted Lithuanian versions of EPDS and IES-R scales were used, with the prior consent of the authors. Other questions were prepared by the publication’s authors, on the basis of the previous inductive qualitative study results.

According to the original EPDS methodology authors, an overall score of 10 on the EPDS scale is considered as a cut-off value indicating increased risk of postnatal depression, and a score above 13 is regarded as an indicator of high risk of postnatal depression [28,29]. However, the information on optimal cut-off scores is inconsistent. The results from the validation study of the Lithuanian version of the EPDS have shown that the EPDS is an optimal screening instrument for severe depressive illness with a cut-off score of 12 and more, yet another Lithuanian study has indicated that the EPDS is a sensitive and accurate instrument for screening for postpartum depressive disorders, with an optimal cut-off score of 7 and more [30,31]. In this study, the original cut-off value of ≥10 was chosen as an indicator of increased risk of postnatal depression, and a cut-off value of ≥12 was selected as an indicator of high risk. In the case of IES-R, the relevant literature has indicated that a mean of ≥1.5 or an overall score of 33 on the IES-R scale is considered as a cut-off value indicating a possible risk of PTSD disorder [32,33].

Data analysis was performed using IBM SPSS Statistics for Windows Version 27.0 (Armonk, NY, USA: IBM Corp.) software. Univariate analysis consisted of the prevalence (n and valid percentages), means, and standard deviations. Missing data accounted for less than 10% of the responses and were inconsistent. Nonparametric analysis criteria were applied because axial variables were not distributed normally based on Kruskal–Wallis, skewness and kurtosis, and histograms. Binary logistic regression in univariate (crude) modeling was applied. Independent variables were demographic and miscarriage-related characteristics, selected on the basis of a two-dimensional statistical analysis; dependent variables—risk of postnatal depression and post-traumatic stress (0—no risk indicated, 1—increased risk indicated). The level of statistical significance was set at *p* < 0.05.

## 3. Results

### 3.1. Miscarriage and Postnatal Depression 

Of the women, 84.7% experienced miscarriage-related tension, 98.7% sadness, and 94.4% feelings of despair, as well as fear of recurrence (93.9%), feelings of confusion (88.0%), mourning (89.7%), emptiness (87.4%), self-blame (79.1%), loneliness (70.0%), helplessness (85.3%), and self-underestimation (67.1%), and had thoughts of self-harm (15.4%) and suicide (14.2%). The mean EPDS score was 11.69 (min 0, max 29, SD 6.24) (n = 839). Of the women, 59.1% were found to be at increased risk of postnatal depression and 48.9% of them at high risk of postnatal depression. 

Binary logistic regression was performed to ascertain the effects of the independent variables—demographic and miscarriage-related characteristics—such as age, education, marital status, type and gestational week of miscarriage, general emotional well-being before pregnancy, emotional and physical well-being immediately after miscarriage, relationship with one’s body, and evaluation of support received, on the likelihood of no risk of postnatal depression in the past 7 days versus increased risk of postnatal depression (dependent variable). The logistic regression model for present postnatal depression risk was statistically significant, X^2^ (9, N = 786) = 168.07, *p* < 0.001. The model explained 25.9% (Nagelkerke R^2^) of the variance in postnatal depression and correctly classified 70.1% of cases.

The results indicated that the women who stated that their relationship with their body after miscarriage was impaired were 2.5 times more likely to experience increased postnatal depression risk compared to those who stated that their relationship with their body was not impaired (OR = 2.48, CI [1.78, 3.44]) (see Table 2). Younger age was associated with an increase in the likelihood of postnatal depression (OR = 1.40, CI [1.01, 1,97]), as well as no higher education (OR = 1.53, CI [1.03, 2.27]), worse emotional well-being before pregnancy (OR = 1.18, CI [1.07, 1.30]), physical well-being immediately after miscarriage (OR = 1.12, CI [1.04, 1.20]), emotional well-being immediately after miscarriage (OR = 1.26, CI [1.12, 1.41]), and less support from family and close friends (OR = 1.15, CI [1.08, 1.23]). Marital status, type of miscarriage, and number of miscarriages and of children born in the family were not associated with postnatal depression risk.

### 3.2. Miscarriage and Post-Traumatic Stress

The mean IES-R score was 31.30 (min 0, max 88, SD 20.22) (n = 839). The mean IES-R avoidance subscale score was 11.90 (SD 7.28), mean intrusion subscale score 12.00 (SD 8.56), and mean hyperarousal subscale score 7.39 (SD 6.50). Of the women, 44.7% were found to be at risk of post-traumatic stress.

Binary logistic regression was performed to ascertain the effects of independent variables—demographic and miscarriage-related characteristics—such as age, education, marital status, number of children in the family, type and gestational week of miscarriage, general emotional well-being before pregnancy, emotional and physical well-being immediately after miscarriage, relationship with one’s body, and evaluation of support received, on the likelihood of no risk of post-traumatic stress in the past 7 days versus increased risk of post-traumatic stress (dependent variable). The logistic regression model for present post-traumatic stress was statistically significant, X^2^ (8, N = 773) = 153.62, *p* = 0.000. The model explained 24.1% (Nagelkerke R^2^) of the variance in postnatal depression and correctly classified 68.3% of cases.

The results indicated that the women who did not have children were 2.5 times more likely to experience increased post-traumatic stress risk compared to those who had 1 child or more (OR = 2.45, CI [1.60, 3.73]) (see Table 3). Those women whose relationship with their body after miscarriage was impaired were 2 times more likely to experience increased post-traumatic stress risk compared to those whose relationship with their body was not impaired (OR = 2.00, CI [1.46, 2.75]). Recurrent miscarriages were associated with an increase in the likelihood of post-traumatic stress risk (OR = 1.17, CI [1.00, 1.37]), as well as with physical well-being immediately after miscarriage (OR = 0.92, CI [1.02, 1.16]), emotional well-being immediately after miscarriage (OR = 1.39, CI [1.22, 1.58]), and insufficient support from family and close friends (OR = 1.13, CI [1.07, 1.20]). Age, educational status, type of miscarriage, and emotional well-being before pregnancy were not associated with postnatal depression risk.

## 4. Discussion

The vast majority of the respondents expressed strong feelings of tension (84.7%), 15.4% have had thoughts of self-harm, and 14.2% of suicide. Of the women, 59.1% were found to be at increased risk of postnatal depression and 48.9% of them at high risk of postnatal depression; 44.7% of women were considered to be at risk of post-traumatic stress. In general, psychological difficulties after miscarriage were found to be particularly common. Although most prior studies report lower rates of depression and PTSD, it is important to note that our study assessed self-reported risk of postnatal depression and PTSS rather than clinically diagnosed disorders [21,22,23]. These results may also be related to the Lithuania-specific sociocultural context and high rates of psychological morbidity in the general population [34].

An impaired relationship with one’s body after miscarriage was the strongest predictor of increased risk of postnatal depression and the second strongest predictor of post-traumatic stress. The role of embodiment in the case of miscarriage has been scarcely explored. However, the self-perceived relationship with one’s body could be the key to a better understanding of the specifics of prenatal loss and trauma. It is hard to differentiate between women’s bodies and a fetus; therefore, they might experience physical emptiness, powerlessness, or loss of control over their reproductive life, which might be one of the key features of pregnancy loss trauma [35]. Furthermore, the literature has demonstrated that prenatal loss characteristics influence the way in which women perceive themselves negatively and affect their general psychological problems [36]. This aspect has been analyzed in another related qualitative research on late miscarriage experiences in a Lithuanian sample. Thematic analysis has described women’s reactions to late miscarriage: the Initial splitting state (Dissociation, An Opened Void, An impaired Symbiosis, and The Body is Still Pregnant while the Psyche is Mourning); Betrayal of the body (Symbolic Experience of Internalized Death, Shocking Materiality of the Ongoing Miscarriage, Lost control of the Body, and Confusing Body Signals); Disconnecting (Depersonalizing Medical Environment, Guilt Falsifies perception, and Retreat as a means of Self-Preservation); and Reconnecting (Collecting Shatters and Reinterpretation of Maternal Identity) [27]. The study mentioned has also revealed that the ability to accept one’s body and the birth of children, as well as the experience of motherhood, were important factors for successful coping after miscarriage. The results differed in terms of support needs from close relatives and friends. Participants in the qualitative study spoke about the need to withdraw themselves from other people as a means of self-preservation, and in the quantitative study, this need for support was especially important. The need to withdraw from contact with others may be associated with poor quality of support, as well as characteristics of late miscarriage.

In our study, childlessness was the strongest predictor of increased risk of post-traumatic stress but was not associated with the risk of depression. This can be explained as a rewriting of one’s trauma through a positive experience that happily completes the narrative of the story. The literature shows that childlessness is an additional stressor for women who have experienced pregnancy loss [37]. Involuntarily childless women report that the greatest fertility-related distress and most reproductive problems are often experienced by them as a significant and chronic stressor [37]. Another study has shown that having children might be associated with a decrease in the symptoms of depression and perinatal grief [14]. 

Younger age, no higher education, worse emotional well-being before pregnancy, worse physical and emotional well-being immediately after miscarriage, and insufficient support from family and close friends were also associated with increased postnatal depression risk. These findings complement and respond to previous studies [12,13,22,23,24]. On the contrary, age, education level, and emotional well-being before pregnancy were not associated with increased risk of post-traumatic stress; however, post-traumatic stress was exclusively associated with recurrent miscarriages. Recurrent miscarriages have been linked with a greater risk of physical complications. They are a risk marker for obstetric complications and placental abruption (including preterm birth and stillbirth in future pregnancies), also serving as a predictor for longer-term health problems, such as cardiovascular diseases and venous thromboembolism [1].

Support from family and close friends was an important predictor in the case of postnatal depression and post-traumatic stress, even though support from healthcare personnel and a partner was associated with neither of the constructs. This result might indicate that support from the outer world (especially support from friends and other women) constitutes an important additional resource. In contrast, partners and medical staff are closely related to the woman who experienced the miscarriage and are, therefore, also affected by the traumatic event. Additionally, Séjourné et al. (2010) emphasized that the most frequently employed coping strategies after miscarriage include discussions with their significant others (86%), participation in internet forums (81%), reading about miscarriage (76%), talking to people from their entourage (70%), talking to other women who had experienced miscarriage (64%), and addressing psychology professionals (15%) [38]. Through contact with the outside world, women look for other perspectives and other sources from where they can obtain necessary information, as well as supportive insights. This confirms the great importance of having a community in difficult times.

## 5. Conclusions

To conclude, the findings support previous research indicating that postnatal depression and PTSS symptoms might persist long after miscarriage, which highlights the importance of paying particular attention to the vulnerability of women after miscarriage and the improvement of post-miscarriage healthcare services [19]. Moreover, this study offers further insight into the role of embodiment and childlessness. Our findings suggest that personalized care and careful screening for psychological morbidity are of great importance. It is essential to include additional social support, especially women’s help to women. However, a great lack of guidelines aiding personnel and public health care professionals, psychologists, and other employees remains. Further research could also help to anticipate and plan preventive measures for women, their partners, and their families after pregnancy loss.

Several limitations of this study have to be mentioned. This study is limited by the use of a single measurement. It is a retrospective self-reported study, so the data are inevitably subjective, and recall bias might influence the results. Furthermore, such a sensitive topic might encourage socially desirable results. The sample is not representative, because it consists of respondents who agreed to participate in the study voluntarily. Such a decision was made taking into consideration the ethical norms so as not to involuntarily stimulate the exposure to the trauma through participation in the study. Thus, a relevant part of the population—women from a lower social class, with lower education, of a single status, with an unplanned pregnancy—might have remained underexplored.

This study has its own advantages. Firstly, its high response rate should be highlighted. Furthermore, validated and reliable instruments to assess the risk of postnatal depression and post-traumatic stress were employed. A wide range of miscarriage cases, such as early/late, single/recurrent, spontaneous miscarriage/partial miscarriage/induced miscarriage, etc., were analyzed. In addition, this study significantly contributes to the specific knowledge base on women’s reproductive health questions. Important, exhaustive new findings might help to better plan screening in order to prevent psychological morbidity after miscarriage.

In future studies, however, we do recommend including a longitudinal aspect. It would help to assess objective changes in well-being and psychological morbidity over time. The needs and coping strategies that lead to better psychological resilience of women after miscarriage are also worth exploring in more depth. Additionally, the sample of male partners and postnatal healthcare professionals has been underrepresented and should also be considered in future studies. Finally, the current study analyzes the well-being of Lithuanian women, but it would be highly beneficial to evaluate the experience of other countries so as to have a better understanding of both local and universal tendencies.

1. Psychological difficulties after miscarriage are common. Of the women, 59.1% were found to be at increased risk of postnatal depression and 48.9% of them at high risk of postnatal depression; 44.7% of the women were regarded as having increased risk of post-traumatic stress.
2. An impaired relationship with one’s body after miscarriage was the strongest predictor for increased postnatal depression risk (OR = 2.48, CI [1.78, 3.44]). Younger age, lack of higher education, worse emotional well-being before pregnancy, worse physical and emotional well-being immediately after miscarriage, and insufficient support from family and close friends were also associated with increased postnatal depression risk.
3. Not having children (OR = 2.45, CI [1.60, 3.73]) and an impaired relationship with one’s body (OR = 2.00, CI [1.46, 2.75]) were identified as the strongest predictors for increased post-traumatic stress risk. Recurrent miscarriages, as well as physical well-being immediately after miscarriage, emotional well-being immediately after miscarriage, and insufficient support from family and close friends, were also linked with an increase in the likelihood of post-traumatic stress.

## Figures and Tables

**Table 1:** Demographic and miscarriage-related characteristics of the study sample (n = 839).

Demographic and Miscarriage-Related Characteristics		n	Valid %
Age	Years, Mean ± SD	Mean 33.34 ± 5.46	
Education	No higher education	204	24.3
	Higher education	635	75.7
Year of the last miscarriage	Min 1993		
	Max 2022		
Marital status	Unmarried	123	14.7
	Married	716	85.3
Number of miscarriages	Single	559	66.6
	Recurrent	280	33.4
Type of miscarriage in medical history	Early (<14 weeks gestation)	737	88.5
	Late (≥14 weeks gestation)	96	11.5
Children born in the family	Doesn’t have children	148	18.3
	Has children	662	81.7

**Table 2:** Binary logistic regression model for present postnatal depression risk.

		B	Wald	Sig.	OR	95% CI for OR	
						Lower	Upper
Age groups	<35/≥35	0.34	4.12	0.042 *	**1.40**	1.01	1.97
Educational status	No higher education/higher education	0.43	4.45	0.035 *	**1.53**	1.03	2.27
Emotional well-being before pregnancy	1–10	0.17	10.49	0.001 *	**1.18**	1.07	1.30
Physical well-being immediately after miscarriage	1–10	0.11	10.04	0.002 *	**1.12**	1.04	1.20
Emotional well-being immediately after miscarriage	1–10	0.23	16.04	<0.001 *	**1.26**	1.12	1.41
Relationship with their body	Was impaired/ was not impaired	0.90	29.26	<0.001 *	**2.48**	1.78	3.44
Support from family and close friends after miscarriage	1–10	0.14	19.89	<0.001 *	**1.15**	1.08	1.23
Type of miscarriage	Early/late	−0.49	3.19	0.074	0.61	0.36	1.05
Marital status	Unmarried/married	−0.42	2.97	0.085	0.66	0.41	1.06

Statistically significant results are presented in bold and marked with * (*p* < 0.05).

**Table 3:** Binary logistic regression model for present post-traumatic stress risk.

		B	Wald	Sig.	OR	95% CI for OR	
						Lower	Upper
Educational status	No higher education/higher education	0.30	2.58	0.108	1.35	0.94	1.96
Born children in the family	Does not have children/has children	0.89	17.20	<0.001 *	**2.45**	1.60	3.73
Number of miscarriages	One/recurrent	0.16	4.05	0.044 *	**1.17**	1.00	1.37
Physical well-being immediately after miscarriage	1–10	0.08	5.72	0.017*	**0.92**	1.02	1.16
Emotional well-being immediately after miscarriage	1–10	0.33	25.62	<0.001 *	**1.39**	1.22	1.58
Relationship with their body	Was impaired/ was not impaired	0.69	18.27	<0.001 *	**2.00**	1.46	2.75
Support from family and close friends after miscarriage	1–10	0.12	17.94	<0.001 *	**1.13**	1.07	1.20
Type of miscarriage	Early/late	−0.47	3.46	0.063	0.63	0.38	1.03

Statistically significant results are presented in bold and marked with * (*p* < 0.05)

## Data Availability

Anonymized databases are available from the corresponding author upon reasonable request.
